# Supplementary material for: Effect of Dam Body Conformations on Birth Traits of Calves in Chinese Holsteins
Source: Animals (Basel). 2023 Jul 9;13(14):2253. doi: 10.3390/ani13142253 (PMC10376613; doi:10.3390/ani13142253)
Supplement: Supplementary file 1 [file animals-13-02253-s001.zip › animals-2452926-supplementary.pdf]

Table S1. The linear translation of body conformation trait into a functional score reference table[1].

Trait	linear scores								
	1	2	3	4	5	6	7	8	9
ST	57	64	70	75	85	90	95	100	95
CW	55	60	65	70	75	80	85	90	95
BD	56	64	68	75	80	90	95	90	85
LS	55	60	65	70	75	80	85	90	95
PS	55	62	70	80	90	80	75	70	65
PW	55	60	65	70	75	79	82	90	95
FA	56	64	70	76	81	90	100	95	85
HD	57	64	69	75	80	85	90	95	100
BQ	57	64	69	75	80	85	90	95	100
SRL	55	64	75	80	95	80	75	65	55
RLRV	57	64	9	74	78	81	85	90	100
UD	55	65	75	85	95	85	75	65	55
MS	55	60	65	70	75	80	85	90	95
FUA	55	60	65	70	75	80	85	90	95
FTP	57	65	75	80	85	90	85	80	75
FUL	50	60	70	80	90	80	70	60	50
RAH	58	65	68	70	75	80	85	90	95
RAW	58	65	68	70	75	80	85	90	95
RTP	57	65	75	80	85	90	85	80	75
ANG	57	64	69	74	78	81	85	90	95

[1] ST, stature; CW, chest width; BD, body depth; LS, loin strength; PS, pin setting; PW, pin width; FA, feet angle; HD, heel depth; BQ, bone quality; SRL, set of rear legs; RLRV, rear leg-rear view; UD, udder depth; MS, median suspensory; FUA, fore udder attachment; FTP, fore teat placement; FUL, fore udder length; RAH, rear attachment height; RAW, rear attachment width; RTR, rear teat placement; ANG, angularity. The different functional scores corresponding to each linear partition of traits are associated with the optimal linear partition when the functional score is 100. It is considered that the Holstein cow's corresponding morphological traits are in their optimal state when the optimal linear partition is achieved.

Table S2. Effects of different body conformation traits on stillbirth[1].

Trait	Level	P value	OR value	95% confidence interval	
				Lower limit	Upper limit
ST	7 points vs. 6 points	0.75	1.53	0.113	20.80
	8 points vs. 6 points	0.71	0.61	0.044	8.36
	9 points vs. 6 points	0.81	1.40	0.089	22.03
CW	5 points vs. 4 points	0.83	0.90	0.337	2.40
	6 points vs. 4 points	0.39	0.41	0.053	3.13
	7 points vs. 4 points	0.61	2.78	0.053	145.81
BD	7 points vs. 6 points	0.55	0.73	0.254	2.08
LS	7 points vs. 6 points	0.24	3.93	0.403	38.26
	8 points vs. 6 points	0.29	4.22	0.299	59.48
PS	3 points vs. 2 points	0.31	0.17	0.005	5.31
	4 points vs. 2 points	0.19	0.10	0.004	2.97
	5 points vs. 2 points	0.24	0.13	0.004	3.87
	6 points vs. 2 points	0.16	0.09	0.003	2.65
PW	7 points vs. 6 points	0.71	0.66	0.07	6.10
	8 points vs. 6 points	0.80	0.75	0.09	6.47
FA	5 points vs. 4 points	0.03	0.04	0.00	0.71
	6 points vs. 4 points	0.11	0.10	0.01	1.67
	7 points vs. 4 points	0.19	0.11	0.00	3.08
HD	5 points vs. 4 points	0.36	3.34	0.25	44.86
	6 points vs. 4 points	0.65	1.89	0.12	30.33
	7 points vs. 4 points	0.81	1.54	0.05	52.03
BQ	7 points vs. 6 points	0.23	0.50	0.16	1.55
	8 points vs. 6 points	0.43	0.18	0.00	12.50
SRL	2 points vs. 1 point	0.03	0.12	0.02	0.83
	3 points vs. 1 point	0.11	0.18	0.02	1.50
	4 points vs. 1 point	0.19	0.21	0.02	2.20
	5 points vs. 1 point	0.35	0.15	0.00	8.54
	6 points vs. 1 point	0.45	5.12	0.07	361.02
RLRV	6 points vs. 5 points	0.92	1.25	0.02	74.90
	7 points vs. 5 points	0.85	1.49	0.02	96.02
	8 points vs. 5 points	0.92	1.25	0.02	100.23
UD	4 points vs. 3 points	0.36	0.37	0.05	3.05
	5 points vs. 3 points	0.27	0.32	0.04	2.47
	6 points vs. 3 points	0.41	0.40	0.04	3.67
	7 points vs. 3 points	0.10	0.06	0.00	1.65
	8 points vs. 3 points	0.67	0.31	0.00	73.71
MS	4 points vs. 3 points	0.87	1.38	0.03	62.63
	5 points vs. 3 points	0.51	3.71	0.07	184.94
	6 points vs. 3 points	0.72	2.05	0.04	98.23
	7 points vs. 3 points	0.33	15.85	0.06	4167.26

FUA	5 points vs. 4 points	0.73	1.59	0.11	22.88
	6 points vs. 4 points	0.56	2.31	0.14	37.81
	7 points vs. 4 points	0.31	5.24	0.21	128.91
	8 points vs. 4 points	0.05	626.55	1.09	359182.88
FTP	5 points vs. 4 points	0.09	0.12	0.01	1.38
	6 points vs. 4 points	0.21	0.14	0.01	3.00
FUL	5 points vs. 4 points	0.36	0.57	0.18	1.87
	6 points vs. 4 points	0.20	0.29	0.04	1.92
RAH	5 points vs. 4 points	0.08	0.05	0.00	1.46
	6 points vs. 4 points	0.03	0.04	0.00	0.76
	7 points vs. 4 points	0.02	0.02	0.00	0.59
	8 points vs. 4 points	0.71	2.47	0.02	311.39
RAW	5 points vs. 4 points	0.28	0.11	0.00	6.04
	6 points vs. 4 points	0.40	0.21	0.01	7.71
	7 points vs. 4 points	0.44	0.23	0.01	9.27
	8 points vs. 4 points	0.97	0.92	0.01	85.20
RTP	6 points vs. 5 points	0.73	1.25	0.35	4.45
	7 points vs. 5 points	0.38	1.92	0.45	8.28
ANG	6 points vs. 5 points	0.40	1.84	0.45	7.45
	7 points vs. 5 points	0.21	0.14	0.01	3.06

[1] ST, stature; CW, chest width; BD, body depth; LS, loin strength; PS, pin setting; PW, pin width; FA, feet angle; HD, heel depth; BQ, bone quality; SRL, set of rear legs; RLRV, rear leg-rear view; UD, udder depth; MS, median suspensory; FUA, fore udder attachment; FTP, fore teat placement; FUL, fore udder length; RAH, rear attachment height; RAW, rear attachment width; RTR, rear teat placement; ANG, angularity; OR, the odds ratio. The lowest linear score collected to produce stillbirth for each conformational trait was used as the reference level for that conformational trait construct. Formulae used in table is following:

$$Logit\ (p) = \ln(\frac{p}{1-p}) = \beta_\gamma + \beta_1 ST_i + \beta_2 CW_j + \beta_3\ BD_k + \beta_4 LS_l + \beta_5 PS_m + \beta_6 PW_n + \beta_7 FA_o + \beta_8 HD_p + \beta_9 BQ_q + \beta_{10} SRL_r + \beta_{11} RLRV_s + \beta_{12} UD_t + \beta_{13} MS_u + \beta_{14} FUA_v + \beta_{15} FTR_w + \beta_{16} FUL_x + \beta_{17} RAH_y + \beta_{18} RAW_z + \beta_{19} RTP_a + \beta_{20} ANG_b$$

Table S3. Effects of different body conformation traits on dystocia[1].

Trait	Level	P value	OR value	95%confidence interval	
				Lowe limit	Upper limit
ST	5 points vs. 4 points	0.10	0.03	0.00	2.04
	6 points vs. 4 points	0.07	0.02	0.00	1.41
	7 points vs. 4 points	0.03	0.01	0.00	0.69
	8 points vs. 4 points	0.08	0.02	0.00	1.50
CW	4 points vs. 3 points	0.63	0.58	0.06	5.29
	5 points vs. 3 points	0.86	1.23	0.13	11.43
	6 points vs. 3 points	0.87	0.83	0.09	7.97
	7 points vs. 3 points	0.73	0.58	0.03	13.36
BD	7 points vs. 6 points	0.99	1.01	0.51	2.00
LS	5 points vs. 4 points	0.16	0.03	0.00	3.97
	6 points vs. 4 points	0.27	0.13	0.00	5.11
	7 points vs. 4 points	0.66	0.46	0.01	15.88
	8 points vs. 4 points	0.78	0.60	0.02	22.21
PS	4 points vs. 3 points	0.70	1.24	0.42	3.62
	5 points vs. 3 points	0.88	1.09	0.36	3.35
	6 points vs. 3 points	0.45	0.62	0.17	2.18
	7 points vs. 3 points	0.32	0.37	0.05	2.64
PW	6 points vs. 5 points	0.01	0.02	0.00	0.43
	7 points vs. 5 points	0.02	0.03	0.00	0.52
	8 points vs. 5 points	0.02	0.03	0.00	0.61
FA	3 points vs. 2 points	0.41	34.41	0.01	163974.75
	4 points vs. 2 points	0.77	0.34	0.00	513.16
	5 points vs. 2 points	0.37	0.03	0.00	52.45
	6 points vs. 2 points	0.39	0.04	0.00	65.60
	7 points vs. 2 points	0.34	0.03	0.00	47.13
HD	5 points vs. 4 points	0.97	1.04	0.18	5.88
	6 points vs. 4 points	0.73	1.38	0.22	8.69
	7 points vs. 4 points	0.54	1.97	0.23	17.12
	8 points vs. 4 points	0.47	3.08	0.15	64.99
BQ	5 points vs. 3 points	0.45	0.21	0.00	11.66
	6 points vs. 3 points	0.11	0.04	0.00	2.12
	7 points vs. 3 points	0.09	0.04	0.00	1.77
	8 points vs. 3 points	0.14	0.04	0.00	3.00
SRL	2 points vs. 1 point	0.61	1.59	0.26	9.67
	3 points vs. 1 point	0.43	2.10	0.34	13.09
	4 points vs. 1 point	0.33	2.61	0.38	18.16
	5 points vs. 1 point	0.79	1.38	0.13	14.88
	6 points vs. 1 poin	0.59	0.16	0.00	127.33
	7 points vs. 1 point	0.69	0.44	0.01	25.98
RLRV	4 points vs. 3 points	0.80	0.57	0.01	43.16

	6 points vs. 3 points	0.47	3.60	0.11	113.52
	7 points vs. 3 points	0.25	7.85	0.23	270.55
	8 points vs. 3 points	0.20	10.31	0.29	366.52
UD	3 points vs. 2 points	0.69	0.22	0.00	350.06
	4 points vs. 2 points	0.48	0.07	0.00	98.96
	5 points vs. 2 points	0.60	0.14	0.00	192.95
	6 points vs. 2 points	0.53	0.10	0.00	137.38
	7 points vs. 2 points	0.35	0.03	0.00	46.15
	8 points vs. 2 points	0.39	0.03	0.00	69.16
MS	4 points vs. 3 points	0.46	0.45	0.05	3.77
	5 points vs. 3 points	0.70	0.65	0.08	5.57
	6 points vs. 3 points	0.57	0.53	0.06	4.65
	7 points vs. 3 points	0.46	2.54	0.21	30.50
	8 points vs. 3 points	0.80	0.64	0.02	19.11
FU	5 points vs. 4 points	0.50	0.57	0.11	2.89
	6 points vs. 4 points	0.16	0.30	0.06	1.61
	7 points vs. 4 points	0.10	0.19	0.03	1.39
	8 points vs. 4 points	0.65	0.41	0.01	20.14
FT	5 points vs. 4 points	0.64	0.71	0.16	3.04
	6 points vs. 4 points	0.43	0.51	0.09	2.72
FU	4 points vs. 3 points	0.49	14.08	0.01	26396.59
	5 points vs. 3 points	0.48	15.09	0.01	27526.28
	6 points vs. 3 points	0.49	14.42	0.01	25976.33
	7 points vs. 3 points	0.27	72.34	0.03	154075.69
RAH	5 points vs. 4 points	0.10	30.30	0.53	1733.03
	6 points vs. 4 points	0.39	5.80	0.11	317.40
	7 points vs. 4 points	0.33	7.54	0.13	428.25
RAW	4 points vs. 3 points	0.15	0.04	0.00	3.10
	5 points vs. 3 points	0.21	0.05	0.00	5.29
	6 points vs. 3 points	0.27	0.08	0.00	7.59
	7 points vs. 3 points	0.32	0.09	0.00	9.87
	8 points vs. 3 points	0.19	0.04	0.00	5.10
RTP	6 points vs. 5 points	0.66	0.84	0.40	1.80
	7 points vs. 5 points	0.78	1.15	0.43	3.05
ANG	5 points vs. 4 points	0.61	0.38	0.01	15.99
	6 points vs. 4 points	0.40	0.20	0.01	8.82
	7 points vs. 4 points	0.26	0.10	0.00	5.43

[1] ST, stature; CW, chest width; BD, body depth; LS, loin strength; PS, pin setting; PW, pin width; FA, feet angle; HD, heel depth; BQ, bone quality; SRL, set of rear legs; RLRV, rear leg-rear view; UD, udder depth; MS, median suspensory; FUA, fore udder attachment; FTP, fore teat placement; FUL, fore udder length; RAH, rear attachment height; RAW, rear attachment width; RTR, rear teat placement; ANG, angularity; OR, the odds ratio. The lowest linear score collected to produce stillbirth for each conformational trait was used as the reference level for that conformational trait construct.

Table S4. The mean gestation length (d) by linear score for each body conformation trait[1].

Trait	1	2	3	4	5	6	7	8	9	P value
ST				276.50±5.50	280.33±4.33	273.17±1.90	273.06±1.20	273.02±0.84	273.93±0.77	0.87
CW			273.70±2.83	273.53±0.79	273.09±0.82	274.13±1.25	272.80±3.17	251.50±22.50		0.19
BD				251.50±22.50	278.22±1.46	273.18±0.97	273.51±0.59	270.29±6.80		0.06
LS				278.00±3.22	274.18±2.23	274.23±0.97	272.81±0.71	273.81±1.05		0.74
PS		271.25±5.19	274.72±1.34	273.53±0.96	273.61±0.81	272.78±1.19	272.83±2.26	250.00±22.00		0.17
PW				258.50±30.50	272.54±2.70	273.51±0.85	273.19±0.69	275.53±0.90		0.33
FA		279.75±3.73	273.11±2.25	271.78±2.80	273.62±1.08	274.18±0.59	271.66±1.41	267.33±1.41		0.31
HD			269.14±7.42	273.72±2.70	274.25±0.69	272.97±0.87	272.59±1.23	275.04±1.17		0.71
BQ		269.00±13.00	264.00±7.15	274.80±3.79	271.57±2.52	272.71±0.77	274.46±0.72	273.81±2.71		0.37
SRL	264.33±2.32	273.89±0.86	274.01±0.76	272.97±1.22	273.73±1.82	270.83±8.02	271.50±3.29	263.50±7.50		0.10
RLRV		277.75±3.90	273.40±1.40	276.23±1.22	268.58±3.23	272.32±1.15	273.42±0.85	274.08±0.85	281.50±0.71	0.43
UD		277.00±0.58	272.20±3.04	274.12±0.98	272.64±0.85	273.94±0.84	275.13±1.61	273.38±2.17		0.82
MS			275.06±2.99	273.56±0.83	273.60±0.84	272.58±1.10	274.36±1.44	275.00±2.39		0.91
FUA		278.50±1.50	273.50±0.50	274.70±1.59	273.53±0.91	273.28±0.74	272.91±1.31	272.50±4.05		0.99
FTP			268.67±10.93	274.67±1.17	272.96±0.61	274.93±0.80	280.25±2.36			0.40
FUL			274.00±1.00	272.51±0.77	274.24±0.79	273.05±1.52	280.10±1.41			0.21
RAH				269.35±2.83	274.63±0.92	272.65±0.83	274.35±0.76	267.50±5.65		0.12
RAW			273.60±1.01	273.90±2.94	274.13±1.35	273.34±0.80	272.92±0.67	274.91±1.80		0.96
RTR				278.75±1.38	274.04±0.74	272.81±0.82	272.52±1.35	278.40±3.79		0.48
ANG				273.80±5.49	273.20±1.05	273.02±0.72	274.81±0.64	279.00±3.00		0.74

[1] ST, stature; CW, chest width; BD, body depth; LS, loin strength; PS, pin setting; PW, pin width; FA, feet angle; HD, heel depth; BQ, bone quality; SRL, set of rear legs; RLRV, rear leg-rear view; UD, udder depth; MS, median suspensory; FUA, fore udder attachment; FTP, fore teat placement; FUL, fore udder length; RAH, rear attachment height;

RAW, rear attachment width; RTR, rear teat placement; ANG, angularity. There have no cows with the linear score for the trait when cells are blank. This table employs Duncan's multiple range test.
